# The moderating role of underlying predictors of survival in patients with brain stroke: a statistical modeling

**DOI:** 10.1038/s41598-020-72814-w

**Published:** 2020-09-28

**Authors:** Nasrin Someeh, Seyed Morteza Shamshirgaran, Farshid Farzipoor, Mohammad Asghari-Jafarabadi

**Affiliations:** 1 Student Research Committee, Tabriz University of Medical Sciences, Tabriz, Iran; 2 Department of Statistics and Epidemiology, Faculty of Health Sciences, Neyshabur University of Medical Sciences, Neyshabur, Iran; 3 Department of Health Education and Promotion, Faculty of Health, Tabriz University of Medical Sciences, Tabriz, Iran; 4 Department of Statistics and Epidemiology, Faculty of Health, Tabriz University of Medical Sciences, PO. Box: 5166614711, Tabriz, Iran

**Keywords:** Health care, Medical research, Risk factors

## Abstract

Determining subclinical Brain stroke (BS) risk factors may allow for early and more operative BS prevention measures to find the main risk factors and moderating effects of survival in patients with BS. In this prospective study, a total of 332 patients were recruited from 2004 up to 2018. Cox's proportional hazard regressions were used to analyze the predictors of survival and the moderating effect by introducing the interaction effects. The survival probability 1-, 5- and 10-year death rates were 0.254, 0.053, and 0. 023, respectively. The most important risk factors for predicting BS were age category, sex, history of blood pressure, history of diabetes, history of hyperlipoproteinemia, oral contraceptive pill, hemorrhagic cerebrovascular accident. Interestingly, the age category and education level, smoking and using oral contraceptive pill moderates the relationship between the history of cerebrovascular accident, history of heart disease, and history of blood pressure with the hazard of BS, respectively. Instead of considerable advances in the treatment of the patient with BS, effective BS prevention remains the best means for dropping the BS load regarding the related factors found in this study.

## Introduction

Brain stroke (BS) is among the primary sources of mortality and permanent disability in the world. BS is the second leading cause of death in Iran. Besides, it causes more long-standing severe disabilities than any other disease. More than half of patients with BS will die within the subsequent eight years. An approximately three-quarters of all strokes arise in the community over the age of 65. The risk of developing BS would double more than per decade after the age of 55 (U.S. Centers for Disease Control and Prevention). According to the World Health Organization^1^, 15 million people suffer from BS worldwide each year. Of these numbers, 5 million dies, and the other 5 million are permanently disabled. High blood pressure contributes to more than 12.7 million strokes worldwide. Europe averages approximately 650,000 BS death each year. These figures indicate the importance of studying BS.

Modifiable factors to support endorse survival among patients with BS are of central public health importance. Hypertension is the most potent risk factor for BS. Age and the existence of other risk factors may alter or improve the result of increased blood pressure on BS incidence 2–5. According to the common risk factors among patients with BS, in developed countries, the incidence of stroke is declining, mainly due to efforts to lower blood pressure and reduce smoking. However, the overall rate of stroke remains high due to the aging of the population. Impaired cardiac function growths stroke incidence at all levels of blood pressure^2,3^. It has been suggested that the history of hyperlipoproteinemia is a risk factor for BS and that the increased stroke incidence in persons with long term exposure to the risk factors promotes atherosclerosis and subsequently develops BS Diabetes increases the risk of cardiovascular and cerebrovascular diseases (such as ischemic BS)^4^. Around 30% of BS patients have diabetes^4^ neurological deficits and death rates are meaningfully higher in patients with BS who also have diabetes ^5^. Smoking and passive smoking, as one of the main risk factors for BS, has been identified by some studies^6,7^ passive smoking increases the risk of overall BS 30%^7^. Determining BS risk factors may allow for early and possibly more operative BS prevention.

Based on an extensive search, no study was found (if any) to model the risk factors of the hazard of death and their possible moderating effects among patients with BS in Ardabil, Iran. Therefore, this study aimed to model the main predictors of survival in patients with BS, along with the moderating effects of these factors in a period of 10-years follow up.

## Materials and methods

### Study design and procedure

In the current prospective longitudinal study, data were acquired from the BS registry of the Imam Khomeini Hospital, Ardabil, Iran. All patients complied with the International Coding System ICD-10 according to the Computerized Tomography (CT) scan and Magnetic Resonance Imaging (MRI). A total of 332 patients were entered in the ten-year follow-up study (defined as the date of diagnosis from June 2004 up to June 2018). The follow-up time was calculated from the date of hospitalization by acute BS until the death or end of follow-up, whichever came first. Telephone interviews managed post-BS death outcomes only through one of the main authors/researchers (FF) of the study. An exact date of death and cause of death were recorded, except for cases that did not receive a response after multiple follow-ups, and those who died due to non-BS.

### Main variables and measures

For all patients and based on hospital document, the demographic variables included age category at diagnosis (1: >  = 58; 2: 59–68; 3: 69–75; <  = 76), sex (1: male; 2: female), employment status (1: employed; 2: unemployed), place of residence (1:urban; 2:rural), education level (1: diploma-; 2:academic), smoking (1: yes; 2:no), former smoking (1: yes; 2: no), waterpipe smoking (1: yes; 2: no), history of heart disease (1: yes; 2:no), diabetes (1: yes; 2: no), oral contraceptive pill use (1: yes; 2: no), physical activates (1: yes; 2: no), history of cerebrovascular accident type (1: ischemic; 2: hemorrhagic), history of blood pressure history (1: yes; 2: no), history of hyperlipoproteinemia (1: yes; 2: no), history of myocardial infraction (1: yes; 2: no) were used in the analysis.

### Ethical considerations

The protocol of the study was approved by the institutional review board of Tabriz University of Medical Sciences (ethics code: IR.TBZMED.REC.1398.667). Every person was free to participate in the study, and the privacy of participants was also preserved. All participants filled and signed the consentment and also, informed consent has been taken from all participants. All methods were carried out in accordance with relevant guidelines and regulations.

### Statistical analyses

Statistical analysis was done by STATA software [ver.15] (Stata Corp, College Station, Texas USA). Data were presented using mean (SD), median (min–max) for the numeric normal and non-normal variables respectively, and frequency (percent/rate) for categorical variables. Survival time was calculated in months and was expressed as the median (0.95% confidence interval (CI)). Data were described with frequency, rate, and proportion. The Kaplan–Meier method was used to estimate the rates of BS The survival probabilities were compared in groups by the Log-rank test. Cox proportional hazards regression analysis has been conducted to determine the relationship among related BS risk factors, by estimation of its hazard ratio (HR) and 90% CI. In the multivariate analysis, a backward elimination strategy with (*p* < 0.1) was utilized. Adjustments were made for background variables including age category, sex, employment status, place of residence, education level and physical activity for the interested BS risk factors including history of hypertension, history of diabetes, history of cerebrovascular accident, history of myocardial infraction, history of blood pressure history, history of heart disease, history of hyperlipoproteinemia history, smoking, oral contraceptive pill use, and cerebrovascular accident type. Also to assess the moderating effect, the interaction effect of the risk factor of BS with background variables were tested in the model^8^.

Furthermore, Harrell's C index was computed as a measure of concordance between model predictions and real outcomes (Harrell et al., 1984). The missing data ranged over 0.33%—2.01% which were ignorable and were deleted listwise in the analysis. The Proportional Hazard (PH) assumption was assessed by Shoenfield residuals' test^8^ which indicated that the assumption was satisfied in the global test (P = 0.085) and all individual variables tests (All *p* > 0.05).

### Ethics approval and consent to participate

The study protocol was approved by the institutional review board of Tabriz University of Medical Sciences (IRB No.: TBAMED.REC. 1397.286).

## Results

Of the 480 patients enrolled in this study, 332 were eligible to participate in this study, 32 (13%) persons were censored within 10-years of follow up. The median follows up time was 81.3 (min = 0.0, max = 163.3) months, respectively. The mean age at diagnosis was 77.42 (SD 10.4) months in BS patients. In these patients, 1-, 5- and 10-year survival probability (95% CI) were 68% (63–72), 59% (53—64), 39% (34—45), respectively. The median survival time (95% CI) of these patients was 77.42 (62.07—104.48).

The distribution and comparison of death rate based on demographics of the study participants are presented in Table 1.

**Table 1:** Demographic characteristics of the study participants and the results of log rank test.

Characteristic	N (%)	Number of deaths (%)
**Age category (years)**		
< = 58	88 (26.67%)	29 (32.95%)
59—68	77 (23.33%)	38 (49.35%)
69—75	102 (30.91%)	80 (78.43%)
76 +	63 (19.01%)	50 (79.36%)
**Sex**		
Male	168 (50.60%)	111 (56.00%)
Female	164 (49.40%)	88 (44.00%)
**Employment status**		
Employed	107 (32.23%)	59 (30.00%)
Unemployed	225 (67.77%)	140 (70.00%)
**Education level**		
< = Diploma	322 (96.99%)	195 (98.00%)
Academic	10 (3.01%)	4 (2.00%)
Urban	201 (60.54%)	115 (58.00%)
Rural	131 (39.46%)	84 (42.00%)

Table 2 shows the differences in baseline characteristics between the patients who died within 10-years of follow up and those who survived. Among risk factors: higher age category, being male, having a history of blood pressure, having a history of heart disease, having a history of diabetes, having a history of hyperlipoproteinemia, using oral contraceptive pill (in female patients), having a hemorrhagic cerebrovascular accident, and not being physically active were directly and significantly related to event rate (*p* < 0.05).

**Table 2:** Event rate, per (10,000) and the results of tests compering the rates.

Risk factors	Event rate (95%confidence interval)	*p* Value
**Age category (years)**		
< = 58	27 (19—39)	
59–68	65 (47—89)	**0.003**
69–75	153 (123—190)	** < 0.001**
76 +	204 (155—270)	** < 0.001**
**Sex**		
Female	67 (54—82)	**0.005**
Man	100 (83—120)	
**Physical activity**		
Yes	56 (37—85)	**0.041**
No	86 (74—100)	
**History of myocardial infraction**		
Yes	58 (34—100)	0.188
No	84 (73–97)	
**History of blood pressure**		
Yes	96 (81—115)	**0.005**
No	64 (51–81)	
**Heart disease**		
Yes	101 (78—131)	0.066
No	75 (64—89)	
**History of diabetes**		
Yes	114 (84—154)	**0.027**
No	76 (65—89)	
**History of hyperlipoproteinemia**		
Yes	55 (38—79)	**0.012**
No	89 (76—103)	
**Smoking**		
Yes	79 (57—110)	0.855
No	82 (71—96)	
**Former smoking**		
Yes	84 (58—122)	0.834
No	81 (69—96)	
**Passive smoking**		
Yes	72 (52—100)	0.411
No	84 (72—98)	
**Oral contraceptive pill use** (Just in Females)		
Yes	28 (18—43)	** < 0.001**
No	114 (90—144)	
NA	99 (82—119)	
**Waterpipe smoking**		
Yes	109 (55—218)	0.402
No	81 (70—93)	
**Cerebrovascular accident type**		
Hemorrhagic	118 (88—158)	**0.001**
Ischemic	75 (64—88)	

Bold *p* values indicate significant differences (*p* < 0.1).

NA: not applicable.

## Results of univariate Cox regression

For demographic characteristics, higher age categories and being male were significantly related to death rate (*p* < 0.05). Other factors, i.e., employment status, education, were not associated with the survival probability in patients with BS. (*p* > 0.05). Also, physical activity was inversely and significantly related to the hazard of the event (*p* < 0.05).

Regarding the clinical variables, the result showed that for patients with BS., having a history of diabetes, having hyperlipoproteinemia, using oral contraceptive pill, having hemorrhagic cerebrovascular accident was directly and significantly related to the hazard of the event (All *p* < 0.05), but other clinical variables such as cerebrovascular accident history, myocardial infraction, blood pressure, heart disease, smoking, former smoking, passive smoking, waterpipe smoking were not significantly related to the hazard of the event (*p* > 0.05) (Table 3).

**Table 3:** Results of univariate Cox regression models.

Variables	Hazard ratio	90% CI	*p *Value
**Age category (years)**	1.81 #	(1.61–2.02)	** < 0.001**
< = 58	1		
59—68	2.25	(1.50–3.38)	**0.001**
69—75	4.77	(3.31–6.86)	** < 0.001**
76 +	5.92	(4.00–8.77)	** < 0.001**
**Sex**			
Female	Referent		
Male	1.41	(1.11–1.78)	**0.018**
**Education level**			
Academic	Referent		
Diploma-	1.68	(0.622–4.53)	0.306
**Employment status**			
Employed	Referent		
Unemployed	1.23	(0.91–1.68)	0.177
**Place of residence**			
Urban	Referent		
Rural	1.25	(0.99–1.60)	0.116
**Smoking**			
No	Referent		
Yes	0.98	(0.72–1.33)	0.925
**Former smoking**			
No	Referent		
Yes	0.99	(0.70–1.41)	0.981
**Passive smoking**			
No	Referent		
Yes	0.88	(0.65–1.20)	0.511
**Waterpipe smoking**			
No	Referent		
Yes	1.32	(0.73–2.40)	0.439
**Physical activity**			
No	Referent		
Yes	0.65	(0.44–0.95)	**0.064**
**Oral contraceptive pill use (just in females)**			
No	Referent		
Yes	0.31	(0.20–0.47)	** < 0.001**
**History of cerebrovascular accident**			
No	Referent		
Yes	0.83	(0.63–1.11)	0.297
**History of Myocardial infraction**			
No	Referent		
Yes	0.71	(0.44–1.14)	0.240
**History of blood pressure**			
No	Referent		
Yes	1.39	(1.090–1.78)	**0.026**
**History of heart disease**			
No	Referent		
Yes	1.27	(0.98–1.65)	0.126
**History of diabetes**			
No	Referent		
Yes	1.36	(1.02–1.82)	**0.074**
**History of Hyperlipoproteinemia**			
No	Referent		
Yes	0.640	(0.46–0.90)	**0.027**
**Cerebrovascular accident type**			
Ischemic	Referent		
Hemorrhagic	1.49	(1.12–1.97)	**0.020**

Bold *p* values indicate significant differences (*p* < 0.1).

CI: Confidence interval; #: trend estimates.

## Results of multivariate Cox regression

The results of the multivariate Cox regression for patients with BS are shown in Table 4. Based on the results of multivariate analysis, age category, having a history of heart disease, hemorrhagic cerebrovascular accident type was independently and directly related to the hazard of death. Also, the likelihood ratio (LR) test showed a significant contribution of this set of demographic and clinical variables entered in the model (LR Chi-Square = 87.50, df = 7 (Akaike information criterion (AIC) = 1466.21) and *p* < 0.001). Furthermore, Harrell's C index (= 0.71), indicating a fair level of concordance between observed and predicted death in the study sample.

**Table 4:** results of multivariate Cox regression models.

Variables	Hazard ratio	90% CI	*p* Value
**Age category (years)**			
< = 58	Referent		
59—68	2.37	(1.39–4.05)	**0.002**
69—75	6.43	(3.88–10.66)	**<0.001**
76 +	7.35	(4.27–12.64)	**<0.001**
**Heart disease**			
No	Referent		
Yes	1.61	(1.14–2.33)	**0.008**
**Former smoker**			
No	Referent		
Yes	0.72	(0.47–1.11)	0.133
**Cerebrovascular accident type**			
Ischemic	Referent		
Hemorrhagic	2.39	(1.60–3.56)	**< 0.001**
**Physical activity**			
Yes	Referent		
No	0.67	(0.41–1.09)	0.107

Bold *p* values indicate significant differences (*p* < 0.1).

### Assessing the moderating effect using interactions

The results to assess the interaction effect of the significant risk factor of BS with background variables are presented in Table 5. The interaction effect of cerebrovascular accident history with age category and education level, the interaction effect of history of heart disease with smoking, and the interaction effect of history of blood pressure with oral contraceptive pill use were significant on the hazard of BS.

**Table 5:** the interaction of major risk factors of BS with background variables.

Variables	CVAhis (yes)	Mihis (yes)	BPhis (yes)	Heartdis (yes)	Diabhis (yes)	HLPhis (yes)	CVA (hemorrhagic)
Age category (years)							
–58	Referent	Referent	Referent	Referent	Referent	Referent	Referent
59–68	0.008 (1.51–15.53)*	0.44 (0.12–2.51)	0.65 (0.46–3.47)	0.37 (0.58–4.19)	0.52 (0.23–2.09)	0.77 (0.25–2.76)	0.83 (0.31–2.55)
69–75	0.48 (0.49—4.56)	0.73 (0.17–3.45)	0.75 (0.37–2.07)	0.28 (0.24–1.50)	0.23 (0.18–1.51)	0.47 (0.24–1.94)	0.13 (0.80–5.36)
76 +	0.75 ( 0.22–2.95)	0.99 (0.18–5.60)	0.63 (0.49–3.21)	0.95 (0.36–2.59)	0.07 (0.12–1.10)	0.35 (0.53–6.23)	0.29 (0.19–1.64)
Sex (male)	0.69 (0.44–1.73)	0.55 (0.22–2.24)	0.35 (0.72–2.45)	0.49 (0.42–1.51)	0.31 (0.71–2.88)	0.71 (0.50–2.74)	0.86 (0.54–2.08)
Education level (academic)	0.005 (2.45–134.04)*	NA	0.45 (0.25–23.66)	NA	0.12 (0.67–36.31)	0.55 (0.20–20.2)	0.45 (0.42–4.09)
Employment Status (unemployed)	0.79 (0.42–1.92)	0.83 (0.34–3.86)	0.47 (0.42–1.49)	0.48 (0.61–2.84)	0.07 (0.21–1.07)	0.58 (0.29–1.97)	0.09 (0.90–3.86)
	0.43 (0 .37–1.53)	0.39 (0.52–5.26)	0.33 (0.74–2.42)	0.40 (0.40–1.44)	0.22 (0.77–3.12)	0.28 (0.69–3.59)	0.53 (0.41–1.55)
Smoking (yes)	0.20 (0.21–1.39)	0.54 (0.06–4.14)	0.72 (0.41–1.84)	0.04 (1.02–4.98)*	0.09 (0.87–6.68)	0.52 (0.47–4.46)	0.59 (0.52–3.19)
Former smoking (yes)	0.69 (0.22–2.74)	0.91 (0.22–5.31)	0.41 (0.61–3.40)	0.47 (0.58–3.29)	0.85 (0.36–3.49)	0.52 (0.47–4.46)	0.47 (0.21–2.05)
Passive Smoker (yes)	0.89 (0.45–2.48)	0.46 (0.38–8.49)	0.76 (0.45–2.95)	0.91 (0.47–2.35)	0.94 (0.44–2.42)	0.80 (0.45–2.8)	0.61 (0.53–2.93)
Oral Contraceptive Pill use (yes)	0.30 (0.61–4.91)	0.12 (0.75–12.81	0.03 (0.13–0.91)*	0.92 (0.37–2.96)	0.22 (0.15–1.53)	0.68 (0.25–2.46)	0.63 (0.43–3.98)
Physical Activity (yes)	0.10 (0.02–1.36)	0.72 (0.27–6.48)	0.84 (0.42–2.88)	0.31 (0.18–1.73)	0.41 (0.55–4.24)	0.33 (0.55–5.72)	0.51 (0.21–2.16)
Waterpipe Smoking (yes)	0.60 (0.15–2.96)	0.88 (0.13–10.45)	0.37 (0.12–2.20)	NA	0.20 (0.56–14.95)	0.76 (0.28–5.59)	0.06 (0.97–18.58)

* Bold fonts indicate significant relationships (*p* < 0.1).

NA: not applicable.

CVAhis = history of cerebrovascular accident history, MIhis = history of myocardial infraction, BPhis = history of blood pressure, Heartdis = heart disease, Diabhis = history of diabetes, HLPhis = history of hyperlipoproteinemia, CVA = cerebrovascular accident type.

**Figures 1:**
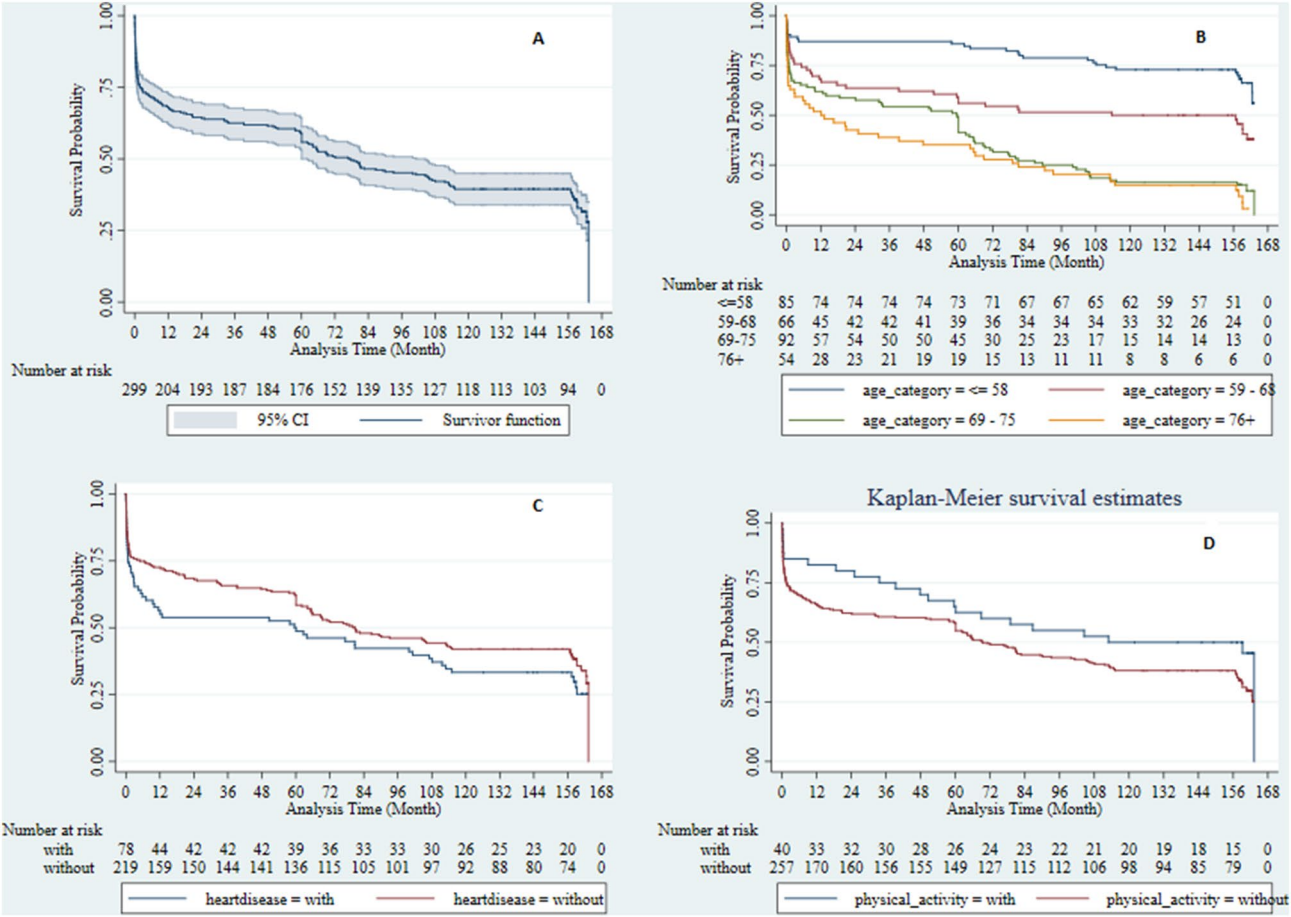
Survival probability of patients with Brain Stroke (BS). (**A**) Overall survival with 95% confidence interval. (**B**) Overall survival by age category. (**C**) Overall survival by the history of heart disease. (**D**) Overall survival by physical activity.

The plot of KM survival probability displays that after 2-months of follow up, the survival rate declined to 75%. Then the mortality rate has dropped steadily until 160 months, and later a sudden fall in survival probability was observed (Fig. 1A). The plot of survival probability by the age category reveals that age category <  = 58 has the highest survival rate among the other age categories, the survival rate for all age categories tends to be similar, and the mortality rate has dropped steadily until 190 months, and then a sudden fall in survival probability was observed (Fig. 1B). Survival probability of patients with BS according to the history of heart disease showed that patients with heart disease have not a better survival prognosis (Fig. 1C). The estimates for patients with BS according to physical activities demonstrate that physically actives patients are survived more than inactive patients (Fig. 1D).

### Assessing the PH assumption

The results of testing the PH assumption indicated that all predictors had satisfied the PH assumption in all cox regression models (*p* > 0.05).

## Discussion

This study was conducted on 332 participants from the Imam Khomeini Hospital, Ardabil, Iran, investigating the predictors and the moderators of survival in patients with BS. The results showed that higher age category, being male, having a history of blood pressure, having a history of heart disease, having a history of diabetes, having a history of hyperlipoproteinemia, using oral contraceptive pill (in female patients), having a hemorrhagic cerebrovascular accident, and not being physically active directly predicted the survival in patients with BS. Interestingly, the interaction effect of cerebrovascular accident history with age category and education level, the interaction effect of history of heart disease with smoking, and the interaction effect of history of blood pressure with oral contraceptive pill use were significant on the hazard of BS, indicating that age category and education level moderates the relationship between the history of cerebrovascular accident and hazard of BS, smoking moderates the relationship between a history of heart disease and hazard of BS, oral contraceptive pill use moderates the relationship between a history of blood pressure and hazard of BS.

The results of univariate analysis for demographic characteristics showed that the age category and sex of the participants were prognostic factors of BS Although, the sex wasn't significant in multivariate analysis. Still, the male patients were more likely to die from BS than the female patients (about 40%). Although the finding of some studies confirms this result^9^, however, some controversies could be explained so that confounders have not been adjusted, and also, women were older when they get their first BS^10^. Other demographic characteristics, i.e., employment status, place of residence, and education level, weren't the significant predictors of survival for BS. Physical activity was a significant prognostic factor of BS based on the results of univariate and multivariate analysis. BS patients with physical activity had a 0.35% hazard rate of less than inactive patients. There is controversy so that Willey et al. reported that any physical activity was not associated with ischemic BS in univariate or multivariable analyses (AHR: 1.16, 95% CI 0.88–1.51)^11^. Differences in methodology, study populations, and interferences are expected to have contributed to these dissimilarities.

Based on the results of univariate analysis for clinical characteristics, having the history of blood pressure was a significant prognostic factor of BS Patients with a history of blood pressure had a worse outcome. Still, it wasn't statistically significant in multivariate analysis. Some studies support this claim^12,13^. History of diabetes was a significant risk factor of BS in univariate analysis. Though it wasn't statistically significant in multivariate analysis, however, a suggestive effect of this variable has been observed so that diabetes develops BS 29% more likely than non-diabetes^3^, Ji et al. confirms the same result^3^. Having the history of hyperlipoproteinemia was a significant risk factor of BS in univariate analysis. Patients without a history of hyperlipoproteinemia develop BS about 36% more than patients with a history of hyperlipoproteinemia, there is a finding in line with our study^2^. Using the oral contraceptive pill was a significant risk factor of BS in female patients in the univariate analysis, the same finding has been assessed from the studies^4,5^. Oral contraceptive pill users were at increased risk of myocardial infarction or BS compared with non‐users: relative risk (RR) 1.6 (95% CI = 1.3‐1.9)^5^. The overall summary odds ratio for first-ever ischemic stroke risk associated with current oral contraceptive pill use compared with noncurrent oral contraceptive pill use was 2.47 [95% CI = 2.04–2.99]. Hemorrhagic cerebrovascular accident was significant for BS according to the results of univariate and multivariate analysis. However, BS patients with hemorrhagic cerebrovascular accident had 1.5 times worse outcomes than those with Ischemic. There are finding that confirms the results of our study^5^.

### Strengths and limitations

The strength of this study is the prospective setting; original features are the instant evaluation of the effect of risk factors on outcomes, allowing assessment of the relative impact of each predictor of survival. However, there are some limitations to this study, and the results should be concluded considering these limitations. Firstly, there may be some evidence of inaccurate responding of the person who accompanied the patients with severe/critical conditions. As the second limitation, some cases of BS immediately lead in death at the time they had been admitted to the hospital that was not included in the study. Thirdly, we hadn’t the NIHSS at baseline in our database as NIHSS may improve the findings and make them more conclusive. Fourthly, we used a simple procedure for follow up over telephone, and there were neither reliability criteria for this nor additional technical/medical work-ups. The telephone-based follow-up is a regular task in survival studies^14,15^. Fifthly, regular care, living conditions, and the quality of receiving essential care couldn't be considered in the assessments. To implement the concerns as a bias in the statistical models, introducing frailty in the model to account for unobserved heterogeneity can be recommended. Such models, along with the cox model, require some assumptions like PH and the independent censoring, which can be relaxed by statistical learning procedures. In this regard, we recommend the deep learning of artificial neural networks as our future program.

## Conclusions

According to the results, age category, sex, employment status, place of residence, education level, physical activity, history of blood pressure, history of diabetes, history of hyperlipoproteinemia, oral contraceptive pill use, cerebrovascular accident were significant risk factors of BS. Also, the age and education, smoking, and using oral contraceptive pill moderates the relationship between cerebrovascular accident history, history of heart disease, and blood pressure history with the hazard of BS, respectively. According to the results obtained in this study, recognizing the BS risk factors provide early and probably more effective BS prevention procedures. Rather than substantial advances for the treatment of a patient with BS, operative BS prevention remains the best action for reducing the BS burden. Also, as modifiable risk factors, increasing physical activity and smoking cessation, the control of high blood pressure, preventing diabetes, and hyperlipoproteinemia may be useful in decreasing the risk of BS for different ages and sexes.
